# Soft skills in radiology: the human core of a technological specialty

**DOI:** 10.1093/bjro/tzag016

**Published:** 2026-07-29

**Authors:** Federico Bruno, Alessandra Splendiani

**Affiliations:** Department of Biotechnological and Applied Clinical Sciences, University of L’Aquila, 67100, L'Aquila, Italy; Department of Biotechnological and Applied Clinical Sciences, University of L’Aquila, 67100, L'Aquila, Italy

**Keywords:** soft skills, radiology education, communication, empathy, leadership

## Abstract

Radiology is often viewed as a technology-driven specialty, yet radiologists work in a highly interactive clinical environment where interpersonal and cognitive competencies are essential. Soft skills (especially communication, empathy, teamwork, adaptability, and leadership) shape patient trust, support effective procedure counseling, and strengthen collaboration with referring clinicians and multidisciplinary teams. Clear, timely, and consultative communication extends radiology’s impact beyond the written report, while responsible digital engagement can enhance professional identity and public trust. As artificial intelligence increasingly automates repetitive imaging tasks, uniquely human strengths such as judgment, ethical reasoning, empathy, and leadership will become even more central to radiologists’ value. Investing in mentorship and structured soft-skills development can improve patient experience, departmental efficiency, and professional resilience, reaffirming the human core of a technological specialty.

In the business and corporate world, soft skills have become central to evaluating candidates for professional positions. Unlike hard skills (the technical competencies acquired through education and training) soft skills encompass interpersonal, behavioral, emotional, and cognitive abilities shaped by personal experiences and professional development. These competencies include, e.g., communication, teamwork, adaptability, empathy, leadership, emotional intelligence, professionalism, and conflict management. In medical education and healthcare literature, related terms such as “interpersonal competencies,” “humanistic competencies,” and “non-interpretive skills” are often used to describe overlapping dimensions of these non-technical attributes. In this manuscript, the term “soft skills” is used as an umbrella concept encompassing these competencies within radiologic practice.^1^^,^^2^

Radiology has traditionally been perceived as a technically oriented specialty centered on advanced imaging technologies and diagnostic interpretation. However, the radiologist’s role is inherently interactive. Radiologists engage daily with patients, referring clinicians, technologists, nurses, and multidisciplinary teams while managing workflow pressures, rotating shifts, and high-stakes clinical decisions. In this context, technical excellence alone is insufficient. Communication, leadership, teamwork, and adaptability significantly influence professional performance, workplace dynamics, and the broader perception of radiologists within healthcare systems.

## Communication and patient-centered care

Communication is one of the most essential soft skills in radiology. Although the radiology report remains the specialty’s primary professional output, radiologists’ professional identity should extend beyond written interpretations. Effective communication enables radiologists to contribute actively to clinical decision-making, imaging selection, protocol optimization, and multidisciplinary discussions. For interventional radiologists, communication is equally important when explaining procedures, managing expectations, and addressing patient concerns with clarity and empathy.^3^

Effective communication is actually essential not only during interventional procedures. Explaining to patients the examination they are about to undergo, whether MRI, CT, ultrasound, or X-ray, helps reassure them and enables them to understand the risks and benefits of the diagnostic investigation, including issues related to exposure to ionizing radiation or the administration of contrast agents. It also helps patients appreciate how the information obtained from the examination may influence their therapeutic pathway, particularly in vulnerable settings such as in emergency settings, oncology patients, frail individuals, older adults, and children. Although the degree of direct patient interaction varies across subspecialties and practice settings, interpersonal competencies remain essential throughout radiologic practice, including communication with referring clinicians, trainees, multidisciplinary teams, technologists, and patients.^4^^,^^5^ Furthermore, clear, transparent, and accessible communication can strengthen trust and reduce the risk of medico-legal disputes.

Communication in radiology may also involve emotionally demanding interactions, including discussion of serious or life-changing findings. Repeated exposure to these situations may contribute to emotional stress, communication avoidance, or professional fatigue. Structured communication training may therefore improve not only patient experience, but also clinicians’ resilience, confidence, and emotional awareness. In this context, communication training should not be viewed solely as a professional requirement, but also as an important component of clinician well-being and sustainable medical practice.

Contemporary healthcare is progressively shifting from a disease-centered model focused on “what is the matter with the patient?” toward a more patient-centered approach that considers “what matters to the patient.” Patients increasingly expect to be informed, educated, and actively involved in decision-making processes.^4^ In this context, active engagement with patients advocacy groups represents an important added value for radiologists, providing a deeper understanding of patients’ perspectives, needs, and expectations.

Radiology also plays a central role in prevention and population-based screening programs. Breast imaging represents an important model of patient-centered radiologic practice in which communication, trust, education, and longitudinal patient engagement are fundamental components of care. Successful implementation of screening strategies depends not only on diagnostic accuracy, but also on patients’ understanding, confidence, and willingness to participate in preventive pathways.

Communication with referring clinicians is equally important. Understanding the clinical question, and conveying results in a timely and actionable manner reinforces radiology’s role as a consultative specialty rather than a purely interpretive service.

Therefore, good communication and empathy skills should be considered not merely desirable or optional qualities for a good radiologist, but core professional competencies that should be learned and continuously developed throughout both training and professional practice. In the digital era, professional communication increasingly extends into online environments. Ethical and responsible engagement on digital platforms represents an emerging aspect of professionalism and communication competence, contributing to public education, health literacy, and the dissemination of reliable medical information.

## Leadership, teamwork, and education

Leadership and teamwork are vital components of radiologic practice. Radiologists frequently serve as consultants across specialties, coordinate diagnostic workflows, and contribute to(or lead)multidisciplinary groups. Leadership in radiology extends beyond formal administrative roles and includes the ability to foster collaboration, inspire trust, manage conflict, and integrate diverse clinical perspectives in the service of patient care.^6^

Multidisciplinary team meetings and tumor boards represent one of the clearest examples of the evolving role of radiologists within modern healthcare systems. In these settings, radiologists increasingly contribute as clinical consultants and coordinators of diagnostic and therapeutic decision-making. Effective participation in multidisciplinary discussions requires clarity, conciseness, professional assertiveness, adaptability, and leadership skills.

Soft skills are also fundamental to the educational and consultative role of radiologists. Radiologists contribute extensively to the education of residents, medical students, technologists, and referring clinicians. Teaching in multidisciplinary settings, explaining imaging findings, discussing clinical cases, and guiding imaging appropriateness all require effective interpersonal communication and empathy.^7^

Despite their partly innate component, soft skills can be strengthened through training and practice. Radiology societies and residency programs increasingly invest in non-interpretive skills education through workshops, communication training, leadership curricula, and mentorship initiatives. Communication workshops, simulation sessions, and role-play exercises based on everyday radiology scenarios may provide safe environments for practicing difficult conversations, informed consent, and patient-centered communication.^8^

Role modeling and observational learning remain central components of soft-skill development. Observing how experienced radiologists conduct difficult conversations, lead meetings, communicate with clinicians and patients, or manage collaboration provides powerful examples of professional behavior and interpersonal competence. Formal mentorship programs, meanwhile, provide structured longitudinal relationships through which trainees receive guidance, feedback, professional support, and career development.^7^^,^^9^ A notable example is the Athena Mentorship Program endorsed by the ESOR, aiming to support mid-career female radiologists through online lectures and live meetings focused on leadership and soft skills development.

### The visible radiologist of the future

The importance of soft skills in radiology can also be interpreted within the broader framework of value-based radiology. Contemporary healthcare systems increasingly evaluate radiology not only according to productivity metrics or imaging volume, but also according to the value imaging contributes to patient outcomes and healthcare efficiency. In this context, radiologists create value not solely through image interpretation, but also through communication with referring clinicians, multidisciplinary collaboration, patient engagement, education, and integration within clinical decision-making pathways.^10^^,^^11^

Recent European Society of Radiology position papers emphasize that radiologists are not merely image interpreters, but clinicians who contribute actively to patient care through consultation, communication, research, and leadership. These statements also highlight the importance of counteracting the traditional perception of radiologists as “invisible” or purely technical specialists by promoting the concept of the increasingly “visible radiologist,” actively integrated within patient-centered care pathways.^12^^,^^13^

The increasing integration of artificial intelligence into radiologic workflows may further enhance the importance of uniquely human competencies. While AI has the potential to support image analysis and workflow optimization, skills such as clinical judgment, empathy, ethical reasoning, communication, adaptability, and leadership remain intrinsically human dimensions of medical practice.

Contemporary radiology may therefore be approaching a critical crossroads in which the true value of AI lies not only in increased efficiency, but also in the possibility of reclaiming time for radiologists. An important question for the future of the specialty is whether these technological gains will primarily translate into greater imaging volume or into greater clinical value. AI-supported workflows may offer radiologists more time for task related to human interaction such as multidisciplinary meetings and patient communication.^14^ In this perspective, soft skills become increasingly important not despite technological progress, but precisely because of it. The evolving integration of AI may therefore represent an opportunity to reinforce the professional identity, visibility, and clinical value of radiologists within modern healthcare systems.

Radiology is entering a period of rapid technological and cultural change, but the future of the specialty should not be defined by invisibility. As imaging becomes increasingly complex and AI becomes embedded in clinical workflows, radiologists will remain essential not only as image interpreters, but also as communicators, educators, clinical advisors, and patient-facing physicians. Strengthening communication skills, visibility within multidisciplinary teams, and engagement with patients should therefore be central to radiology training and professional development. The visible radiologist of the future will be one who combines technical expertise with empathy, clinical judgment, and leadership in patient-centered care.
